# A comparative venomic fingerprinting approach reveals that galling and non-galling fig wasp species have different venom profiles

**DOI:** 10.1371/journal.pone.0207051

**Published:** 2018-11-08

**Authors:** Larissa G. Elias, Denise B. Silva, Ricardo Silva, Yan-Qiong Peng, Da-Rong Yang, Norberto P. Lopes, Rodrigo A. S. Pereira

**Affiliations:** 1 Departamento de Biologia, Faculdade de Filosofia, Ciências e Letras de Ribeirão Preto, Universidade de São Paulo, Ribeirão Preto, São Paulo, Brazil; 2 Laboratório de Produtos Naturais e Espectrometria de Massas (LaPNEM), Universidade Federal de Mato Grosso do Sul, Campo Grande, Mato Grosso do Sul, Brazil; 3 Núcleo de Pesquisa em Produtos Naturais e Sintéticos (NPPNS), Faculdade de Ciências Farmacêuticas de Ribeirão Preto, Universidade de São Paulo, Ribeirão Preto, São Paulo, Brazil; 4 Xishuangbanna Tropical Botanical Garden, Chinese Academy of Sciences, Menglun, Mengla, Yunnan, China; Onderstepoort Veterinary Institute, SOUTH AFRICA

## Abstract

The galling habit represents a complex type of interaction between insects and plants, ranging from antagonism to mutualism. The obligate pollination mutualism between *Ficus* and fig wasps relies strongly on the induction of galls in *Ficus* flowers, where wasps' offspring develop. Even though gall induction plays an important role in many insect-plant interactions, the mechanisms that trigger gall formation are still not completely known. Using a fingerprinting approach, we show here that venom protein profiles from galling fig wasps differ from the venom profiles of non-galling species, suggesting the secretion plays different roles according to the type of interaction it is involved in. Each studied cleptoparasitic species had a distinct venom profile, suggesting that cleptoparasitism in fig wasps covers a vast diversity of molecular interactions. Fig wasp venoms are mainly composed of peptides. No low molecular weight compounds were detected by UPLC-DAD-MS, suggesting that such compounds (*e*.*g*., IAA and cytokinines) are not involved in gall induction. The differences in venom composition observed between galling and non-galling fig wasp species bring new perspectives to the study of gall induction processes and the role of insect secretions.

## Introduction

Venoms play an important role in modulating animal interactions with the surrounding environment, usually being involved in defense or prey capture [1]. Venoms commonly consist of a mixture of peptides, proteins and other compounds that are injected in the prey or host and interfere with its vital systems [2–4]. Venom has evolved independently in more than twenty lineages in Metazoa and is produced by exocrine glands located in different parts of vertebrates’ and invertebrates' bodies [5].

In Hymenoptera the venom apparatus is associated with the female ovipositor, and its ancestral function was probably associated with coating of the eggs [6]. However, major shifts in venom function took place throughout the evolutionary history of Hymenoptera, possibly related to the great diversification experienced by the group. Hymenoptera is one of the megadiverse orders in Insecta, which is reflected in the diversity of life histories, including socialisation, phytophagy, parasitoidism and cleptoparasitism [7]. Specifically in parasitoid Hymenoptera, venoms are injected in the host by the female during oviposition, and are related to host manipulation by interfering with its development, immune response and motor control [8]. Such venoms consist of proteins and polypeptides of high molecular weight and of an acidic nature [8,9]. Most of them are hydrolases that rupture cells and tissues, enabling the action of neurotoxic, paralysing, immunosuppressant and cytotoxic components [10,11]. Phenoloxidases and protease inhibitors are also commonly found in parasitoid venoms [8].

In phytophagous insects, venom might play a role in manipulating plant tissues, leading to the induction of a gall [12–14]. Insect gall induction is a rather complex process that leads to the formation of an entirely new structure, which shelters and nourishes a developing larva [15– 17]. Galls may be induced in response to salivary secretion from larvae (*e*.*g*., Diptera: Cecidomyiidae) [18] or to venom injected by the female during oviposition (*e*.*g*., Hymenoptera: Cynipidae and Tenthredinidae) [14,18]. Some phytohormones such as auxin and cytokinins, which stimulate cell growth and division, have been described in larval salivary secretions and in secretions injected by insects during oviposition [14,16]. Moreover, salivary proteins and peptides probably play a role in gall induction since their expression is detected in galled tissues [19,20]. However, the mechanisms leading to gall formation are still poorly understood [17,21].

The composition and functional role of insect venoms are of particular importance for ecological studies of insect-plant communities, as they help understand mechanisms of species interaction and coexistence in the same system. However, the current knowledge in this field is mainly based on specific studies (*e*.*g*., Zhu, Ye & Hu, 2008 [22]; Goeks et al., 2013 [23]), so that a comprehensive comparative approach is limited [24,25]. In this context, we use *Ficus–*associated wasps (Hymenoptera: Chalcidoidea) as a model of a complex insect-plant community with representatives of different life histories (*i*.*e*., cleptoparasites, gallers and parasitoids). Besides encompassing these different strategies, the system includes independent phylogenetic lineages [26], allowing a comparative approach.

Fig wasps use *Ficus* (Moraceae) pistillate flowers as oviposition sites and create a complex microenvironment inside *Ficus* inflorescences, which contain up to a thousand flowers and are nurseries to up to 30 species of invertebrates [27]. Some fig wasps belonging to Agaonidae have an obligate mutualistic relationship with *Ficus* plants. They enter the fig inflorescence (or fig), deposit their eggs in flower ovaries and pollinate some of the flowers. They are among the few organisms adapted to entering the fig and the only ones that are able to perform pollination [27,28] (but see Compton et al., 1991 [29] and Jousselin, Rasplus & Kjellberg, 2001 [30]). Other fig wasp species belonging to Eurytomidae, Pteromalidae, Torymidae and Ormyridae do not enter figs and oviposit from the exterior, inserting their ovipositors through the fig wall. Some of them are able to induce galls, but none performs pollination [31]. Thus, they are called non-pollinating fig wasps (NPFW).

Gall induction may be triggered by the deposition of the wasp’s venom gland secretion during oviposition [32,33]. Indeed, venom glands have very large reservoirs in most fig wasp species [32,34], which is consistent with the important role their secretion may play [32,33]. After oviposition, each flower ovary becomes a gall, initially by an increase in the volume of nucellus and integument cells, which may be followed by abnormal cellularisation of the endosperm [35,36]. These galls can be exploited by non-galling fig wasp species (cleptoparasites and parasitoids), which use the plant tissue or the developing larvae as resources for their offspring [33,37]. Thus, the secretion injected by the female wasp during oviposition might have different functions, *e*.*g*. gall induction or host manipulation.

Cleptoparasitism is a term widely used in fig wasp studies to describe the general strategy of using the gall induced by another galling species (usually the fig pollinators) as resource for offspring development. Cleptoparasites, though phytophagous, are unable to induce galls [33]. Some cleptoparastic species are early-colonising species that oviposit few days or even hours after the galling species [38]. Other cleptoparasitic fig wasps oviposit some weeks after gall induction by the fig pollinator [39]. Therefore, cleptoparasitism in fig wasps encompasses different feeding strategies.

In this study we investigated the venom profile of seven fig wasp species with different life histories (*i*.*e*., gallers, cleptoparasites and parasitoids), encompassing six genera from the main clades of Agaonidae, Sycophaginae and Pteromalidae [26,40]. We used a comparative venomic fingerprinting approach in order to establish a suggestion of relationship between venom composition and function according to different life histories. Specifically we aimed (1) to elucidate differences among fig wasps venom fingerprints, using MALDI-TOF-MS (Matrix Assisted Laser Desorption/Ionization time-of-flight Mass Spectrometry), according with their life histories; and (2) to investigate the presence of low molecular weight components commonly described to participate in gall induction (*e*.*g*., phytohormones) in the venoms of one galling and one non-galling fig wasp species.

## Materials and methods

### Species and study sites

Wasps were collected from *F*. *citrifolia* Mill. in the Ribeirão Preto campus of Universidade de São Paulo, Brazil (21°10′ S; 47°48′ W) and from *F*. *auriculata* Lour., *F*. *hispida* L. and *F*. *semicordata* Buch.- Ham. ex Sm. at the Xishuangbanna Tropical Botanical Garden (XTBG), in Menglun, China (21°41’ N; 101°25’ L) (Table 1). The first author (LGE) was affiliated to Universidade de São Paulo throughout the development of this study and was formally accepted at XTBG to development part of her study under supervision of Dr.Yang-Qiong Peng. Therefore, no other field permission was necessary.

**Table 1:** Life history and taxonomy of the study species.

Wasp species	Subfamily	Life history	Fig host
*Ceratosolen solmsi* Mayr	Kradibiinae	ovary galler^c^	*Ficus hispida*
*Pegoscapus aerumnosus* (Grandi)	Agaoninae	ovary galler^c^	*Ficus citrifolia*
*Idarnes* sp. 3 (*flavicollis* group^a^)	Sycophaginae	ovary galler^d^	*Ficus citrifolia*
*Sycophaga* sp.^b^	Sycophaginae	ovary galler^d^	*Ficus auriculata*
*Idarnes* sp. 1 (*carme* group^a^)	Sycophaginae	cleptoparasite^d^	*Ficus citrifolia*
*Philotrypesis pilosa* Mayr	Sycoryctinae	cleptoparasite^d^	*Ficus hispida*
*Sycoryctes* aff. *trifemmensis*	Sycoryctinae	parasitoid^d^	*Ficus semicordata*

^a^Bouček, 1993 [41]

^b^sensu Cruaud et al. (2011) [26]

^c^ Pollinating species

^d^ Non-pollinating species

The species studied include representatives of ovary galling (two pollinating and two NPFW species), cleptoparasitic (two species) and parasitoid species (one species). Information about each species’ life history was based on literature [33,39,42] or on personal observation by the authors. These species belong to four subfamilies (Table 1 and S1 Fig).

### Chemical analyses of venom gland reservoirs

#### Venomic fingerprinting by MALDI-TOF MS (Matrix-Assisted Laser Desorption/Ionization Time-Of-Flight Mass Spectrometry)

In order to determine the chemical fingerprint of fig wasp venoms, samples were prepared as a pool of five to 10 reservoirs, which were representative of the chemical variation in each species. Samples from *C*. *solmsi* (n = 3 reservoir pools), *Idarnes* sp.1 (n = 2), *Idarnes* sp.3 (n = 2) and *P*. *aerumnosus* (n = 3) were prepared using five reservoirs in each pool (biological replicates) and each sample was analysed in triplicates (technical replicates). Samples from *Ph*. *pilosa* (n = 3 reservoir pools), *Sycophaga* sp. (n = 3) and *S*. aff. *trifemmensis* (n = 3) were prepared using 10 reservoirs in each pool and analysed with replicates when possible. Data in S2 Fig shows spectra obtained from the three samples collected from *P*. *aerumnosus*, which is used here as model species to illustrate variation among samples.

Samples were added to 3 μl of 0.1% TFA and then to a DHB matrix (at 20 mg mL^-1^, prepared with acetonitrile: water with 0.1% TFA 3:7 v/v) at a ratio of 1:1 (v/v). Subsequently, they were homogenised, and 1 μl of the mixture was spotted onto a ground stainless steel MALDI target. For MALDI-TOF MS analysis, acquisitions were performed in positive linear ion mode for two ranges: m/z 1,000–20,000 and 15,000–55,000. The laser frequency of the equipment was set to 1000 Hz, and 3000 shots were averaged for the generation of each mass spectrum. External calibrations were conducted with a mixture of proteins (protein calibration standard I and II of Bruker). Optimization of MALDI-TOF MS methods and stability of reservoirs samples are described in the Supplemental Information (S3 Fig and Supplemental Material and Methods).

#### Gas chromatography-mass spectrometry (GC-MS) analyses of reservoir samples

The GC-MS was used to allow the detection of compounds with low molecular weight, including non-polar and polar compounds e.g. auxins and cytokinins. Samples were analysed by a gas chromatograph directly coupled to a mass spectrometer (SHIMADZU, model GCMS-QP2010) equipped with a DB-5MS chromatography column, using helium as carrier gas at a flow rate of 1 mL min^-1^. The column was initially set at 50°C, then programmed to reach 240°C at a rate of 3°C min^-1^ and held at 240°C for 5 min. Next, the rate was set to 15°C min^-1^ until 300°C, and the temperature was held at 300°C for 5 min. The injection mode was splitless, the injection temperature was 250°C, and the volume of the sample injected was 1 μL.

Samples (n = 2 reservoir pools for each species) were prepared using a pool of 25 venom reservoirs. They were added to 40 μl of solvent (chloroform and hexane at a ratio of 1:1) and sonicated before injection. We analysed venom from one galling (*Idarnes* sp. 3) and one non-galling (*Idarnes* sp.1) species.

#### Liquid chromatography—Mass spectrometry analyses of reservoir samples (UPLC-DAD-MS)

Ultra-performance liquid chromatography coupled to diode array detector and mass spectrometry (UPLC-DAD-MS) was used to allow the detection of compounds with low molecular weight, including non-polar and polar compounds e.g. auxins and cytokinins.

UPLC-DAD-MS analyses were performed using the ACQUITY system (Waters Assoc., Milford, USA) and an ACQUITY C18 BEH (1.7 μm, 2.1 mm × 50 mm) column. The mobile phase applied was acetonitrile (B) and deionized water (A), the column temperature was maintained at 30°C, and temperature in the automatic injection was 10°C. The flow rate and the injection volume were 0.3 mL min^-1^ and 5 μL, respectively, and the applied elution profile was the following: 0–10 min, 10–50% B; 10–10.4 min, 50–100% B; and 10.4–11.2 min, 100% B.

Nitrogen was used as the nebulizing and drying gas and the following parameters were applied: cone voltage of 25 V, capillary voltage of 2.5 KV, extractor voltage of 3.0 V, desolvation gas flow of 650 L h^-1^, desolvation temperature of 350°C, and cone gas flow of 55 L h^-1^.

Samples were analysed using UPLC-DAD-MS in negative and positive ion mode using total ion chromatogram (TIC) and single ion monitoring (SIM) modes. Analyses were focused on ions such as *m/z* 174 [M-H]^-^ (indole-3-acetic acid), 220 [M+H]^+^ (t-zeatin), 204 [M+H]^+^ (isopentenyladenine) and 336 [M+H]^+^ (isopentenyladenosine).

Samples (n = 2 reservoir pools for each species) were prepared using a pool of 10 venom reservoirs. They were added to acetonitrile and deionized water (150 μL) and filtered through Millex filters (0.22 μm, PTFE). We analysed venom from one galling (*Idarnes* sp. 3) and one non-galling (*Idarnes* sp.1) species.

### Data analyses

#### Processing of MALDI-TOF MS spectra for data from venom reservoirs

Venom fingerprints were determined based on MS data of each sample (distribution of peaks for each mass range) obtained by MALDI-TOF MS. All the spectra obtained from linear mode analyses for the seven species studied were externally calibrated in FlexAnalysis software, exported as text files and subsequently imported into the R environment [43]. Spectra were processed using the MALDIquant package [44].

Mass spectra were square-root transformed and smoothed using both Moving Average and Savitzky-Golay methods. Baseline correction was performed using TopHat. Then, mass spectra were aligned and peak detection was performed using a signal-to-noise ratio of 5.

#### MALDI-TOF MS multivariate data analysis

In order to differentiate among the profiles of fig wasps' venom, the intensity matrix for all detected peaks from all samples was used to perform an unsupervised principal component analysis. Analyses were performed in R using "pcaMethods" [45], and "rgl" [46] packages and principal components were displayed in a three-dimensional score plot. After tests of scaling methods listed by van den Berg et al. (2006) [47], we observed that the scale transformation carried out by MALDIquant processing was efficient to reduce within cluster member distances and no additional scaling was applied to the data set.

The same data were also used to perform a supervised analysis, enabling the detection of discriminant peaks (*m/z* values) which have highest separation power regarding ecological groups (pollinating ovary-galling wasps, non-pollinating ovary-galling wasps, cleptoparasites and parasitoids). We used a Partial Least Squares Discriminant Analysis (PLS-DA) with the classical orthogonal score algorithm. The coefficient of multiple determination (R^2^) and cross-validated R^2^ (Q^2^) were used to assess model fitting. Then, a ranking of discriminant peaks was generated using a weighted average of PLS loadings according to Xia et al.(2015) [48]. Analyses were performed in R using the "pls" package [49].

## Results

### Chemical analyses of venom gland reservoirs

#### Venomic fingerprint by MALDI-TOF MS (Matrix-Assisted Laser Desorption/Ionization Time-Of-Flight Mass Spectrometry)

**Fig 1:**
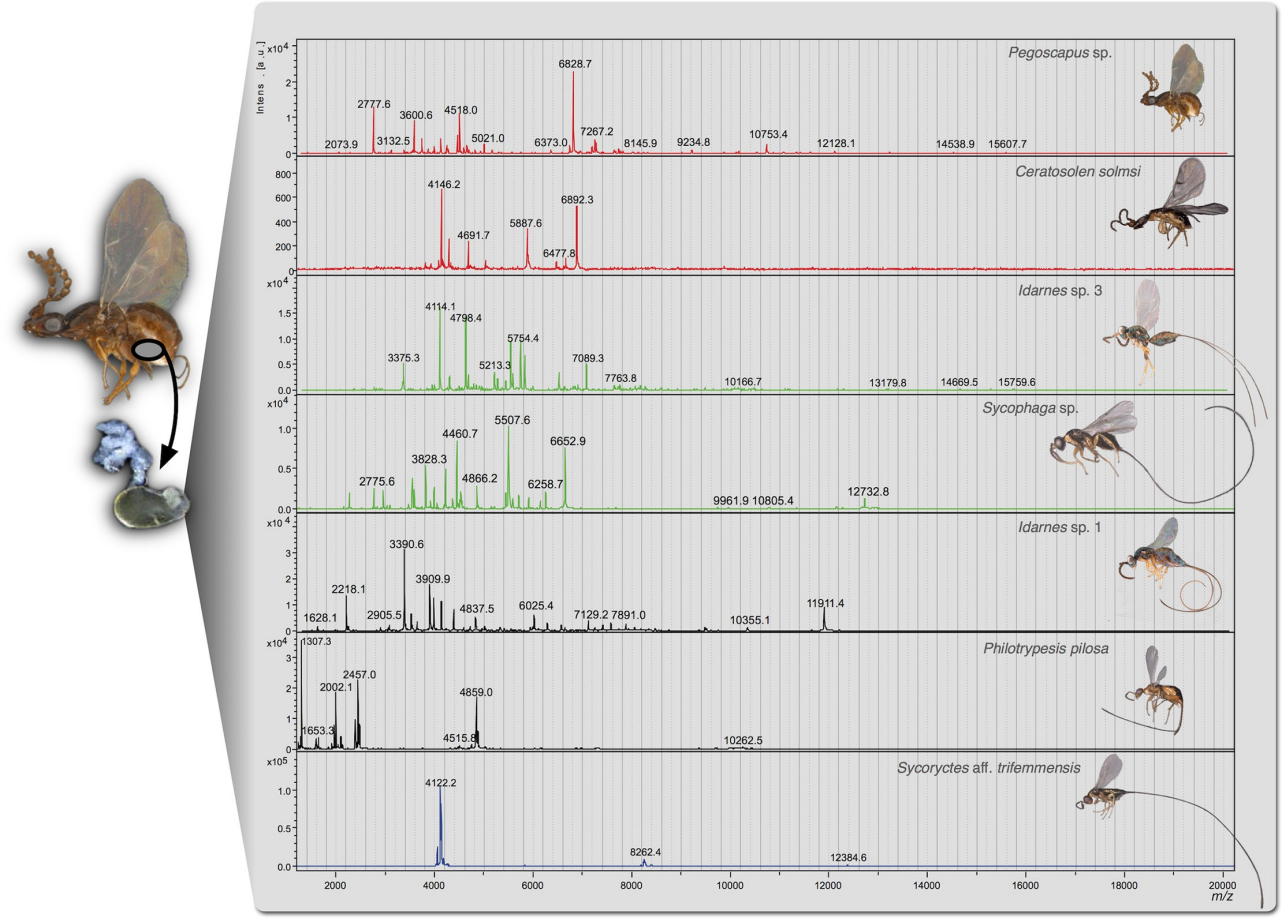
Mass spectra obtained from venom reservoirs using MALDI-TOF MS (linear positive ion mode) in the range *m/z* 1,000–20,000. The image on the left shows a schematic representation of the location of venom gland reservoir in the wasps’ body (grey circle). The arrow points to an image of a dissected venom reservoir. Red spectra correspond to pollinating ovary-galling species, green spectra correspond to non-pollinating ovary-galling species, black spectra correspond to cleptoparasitic species and the blue spectrum corresponds to the parasitoid species.

MALDI-TOF MS analysis of the venom reservoir content of the studied fig wasp species revealed complex mixtures of peptides and proteins with the highest ion intensities within the mass range *m/z* 2400–7000. The venom fingerprint shows that galling and non-galling species have distinct peptide profiles, suggesting that their venoms may differ in composition (Fig 1).

The parasitoid species *S*. aff. *trifemmensis* has the most distinct spectrum among the studied wasps. It is the species with the lowest complexity concerning the compounds with *m/z* 1,000–20,000, including an intense ion at *m/z* 4119.9522, whose mass was accurately determined using reflectron mode analyses (S4 Fig). On the other hand, *Ph*. *pilosa* was the only species that showed a low mass peptide at *m/z* 1307, which represented the highest ion intensity in the range analysed (Fig 1).

**Fig 2:**
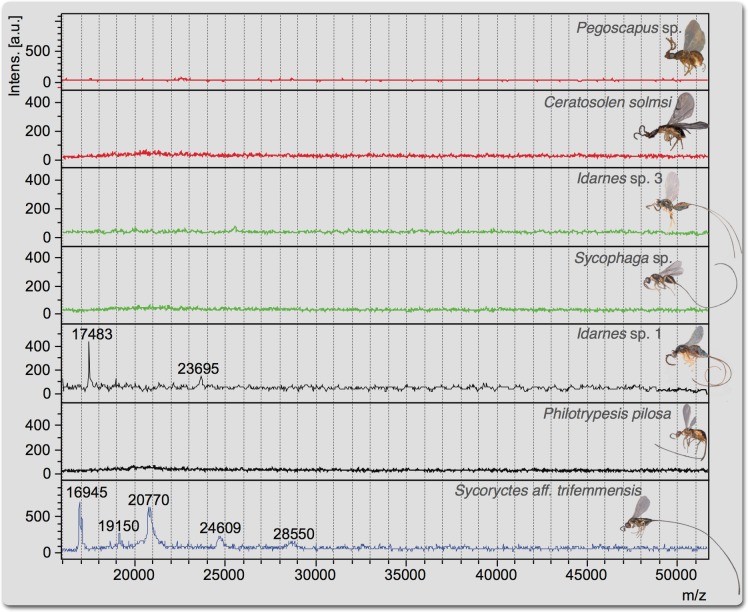
Mass spectra of venom reservoirs obtained using MALDI-TOF MS (linear positive ion mode) in the range *m/z* 15,000–55,000 KDa. Red spectra correspond to pollinating ovary-galling species, green spectra correspond to non-pollinating ovary-galling species, black spectra correspond to cleptoparasitic species and the blue spectrum corresponds to the parasitoid species.

Regarding the range m/z 15,000–55,000, the differences among spectra are striking. *S*. aff. *trifemmensis* is the only species that presents multiple peaks in this range (Fig 2 and S5 Fig). *Idarnes* sp. 1 presents two ions in this range, but the most intense was observed at *m/z* 17483 (Fig 2). However, no peaks were observed in this range for any of the other species.

#### MALDI-TOF MS multivariate data analysis

**Fig 3:**
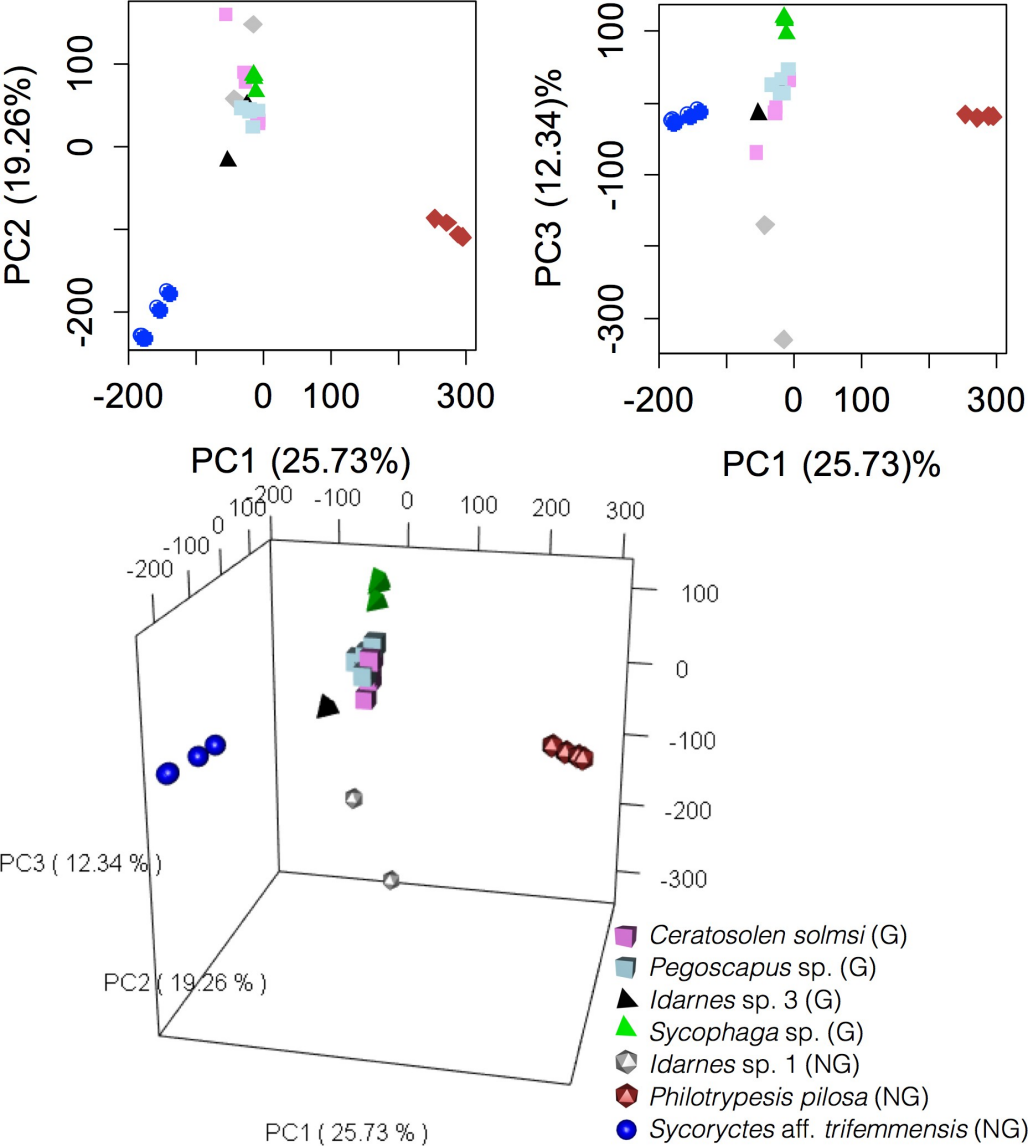
Principal component analysis based on the intensity matrix of ions detected using MALDI-TOF MS at *m/z* 1,000 to 20,000. Top plots represent bidimensional principal components. The bottom plot is a tridimensional representation of principal components 1, 2 and 3. G = galler; NG = non-galler.

Overall, venom spectra at *m/z* 1,000 to 20,000 showed that ovary-galling species (pollinators and non-pollinators) cluster together and are clearly separated from non-galling species (parasitoid and cleptoparasites, Fig 3).

The analysis defined four different groups among the studied fig wasps. The first axis explained 26% of the variation and separated the galling species + *Idarnes* sp. 1 from cleptoparasitic *Ph*. *pilosa* and from the parasitoid *S*. aff. *trifemmensis* (Fig 3). The second axis separates the Pteromalidae species (*Ph*. *pilosa* and *S*. aff. *trifemmensis*) from pollinating + Sycophaginae species, explaining 12% of the variation. The third axis separates the cleptoparasitic *Idarnes* sp. 1 from the gallers. The non-galling species, however, did not form a distinct group (Fig 3).

Regarding cross-validation data for the supervised partial least squares analysis, the accumulated R^2^ and Q^2^ for three components were 0.998 and 0.94, respectively. According to the analysis, the 10 most important ions separating the species according to their life histories were, in this order, *m/z* 4124, 4140, 4068, 1307, 4192, 8260, 2456, 2398, 8245 and 8281 (S6 and S7 Figs).

#### GC-MS and UPLC-DAD-MS analyses of reservoir samples

We did not detect any of the most common auxins and cytokinins nor their precursors, such as indole-3-acetic acid, t-zeatin, isopentenyladenine and isopentenyladenosine, or other ions of low molecular weight, from the venom reservoir content of galling and non-galling species (S8–S13 Figs).

## Discussion

Our results show that MALDI-TOF MS was an adequate tool for differentiating venom fingerprints according to life history. The results also suggest that venom composition is not phylogenetically constrained, probably due to its significant functional role. Indeed, *Idarnes* sp. 1 (cleptoparasite) and *Idarnes* sp. 3 (galler) belong to sister clades in Agaonidae [50], but have different venom compositions, which seem to be related to their different life histories and to venom function.

The venom fingerprints of galling species were more similar to each other than to those of non-galling species. However, initial gall induction strategies might differ among them. For instance, the pollinating *Pegoscapus* larvae depend on endosperm for their nutrition and therefore gall development usually occurs after fertilisation of the flower ovule, and involves cellularisation of the endosperm [35]. On the other hand, non-pollinating *Idarnes* sp. 3 larvae do not rely on endosperm as a resource, and gall induction by this species involves major modifications in nucellus and integument cells [36]. Gall induction by fig wasps is probably related to the venom injected by the female during egg deposition since ovary tissues show anatomical modifications as early as four days after oviposition [35]. Pollinator larvae usually hatch from the eggs two to six days after oviposition, however, sclerotised mouthparts do not develop until the second larval stage (16–18 days after oviposition). At this stage, when larvae start to feed actively and may release salivary secretion, galls are almost completely developed [35], excluding the possibility of a salivary secretion effect, as described for other insects [16,51].

We did not detect any of the most common auxins or cytokinins nor their precursors in any of the analysed reservoir samples of galling and non-galling species, suggesting that the secretion might be involved in signalling molecular and physiological changes in plant tissue rather than directly inducing them. On the other hand, we detected abundant peptides that could be related to gall induction. Some of these peptides were also detected inside the galls, supporting our hypothesis (L.G Elias, unpublished data). However, the role of peptides and proteins in gall formation is still incipient [51], and more information is needed to expand the range of studies about gall formation.

The venom profiles of the two cleptoparasitic species (*i*.*e*., *Idarnes* sp. 1 and *Ph*. *pilosa*) are different from each other, as well as from the other species studied, suggesting that venoms from these species have distinct functions. *Ph*. *pilosa* females oviposit in figs of *F*. *hispida* shortly (one or two days) after oviposition by its host *C*. *solmsi* [52]. At this stage, no macroscopic gall development by *C*. *solmsi* is observed, and secretion from the female *Ph*. *pilosa* may be involved in enhancing gall induction or in delaying development of the host larva. On the other hand, *Idarnes* sp.1 wasps oviposit in figs of *F*. *citrifolia* about 15–20 days after the pollinator oviposition (*i*.*e*., *Pegoscapus* sp.), when galls are fully developed [39,53]. Species belonging to the *Idarnes carme* group are typical representatives of the late-colonising cleptoparasites [53]. The parasitoid strategy is not likely because the host galling larva at this time is not large enough to sustain the *Idarnes carme* group larva. Moreover, it has been demonstrated that *Idarnes carme* group species are indeed phytophagous, as they are able to bore good seeds when there is a shortage of host galls [54]. Thus, these two examples of feeding strategies show that cleptoparasitism involves complex trophic interactions, with particular ecological and developmental implications. Some early cleptoparasitic species [e.g., *Diaziella yangi* and *Lipothymus* sp. (Pteromalidae)] are even described as secondary gallers, as they independently stimulate additional gall growth [55]. Therefore, the venoms of *Idarnes* sp.1 and *Ph*. *pilosa* probably play different roles. *Idarnes* sp1 venom is not related to gall formation and is probably involved in interaction with the host larva. Indeed, at this stage, the host larva is in the second or third larval instar and has already developed mouthparts [35]. Venom might be related to paralysis or developmental arrest of the host larva, allowing the cleptoparasitic larva to compete for resources in the gall. The presence of two ions at *m/z* higher than 17,000 in *Idarnes* sp.1 venom support this hypothesis since higher molecular weight proteins are normally characteristic of parasitoid species [9], which manipulate the host larva. Very little is known about the physiological basis of interaction between cleptoparasites and their hosts, and our results suggest that the definition of cleptoparasitism covers a vast diversity of molecular interactions.

The parasitoid *S*. aff. *trifemmensis* presented the most distinct venom profile among the species studied. This was the only species with multiple proteins in the range of *m/z* 15,000–55,000, corroborating data for other parasitoid species that, in general, present large proteins in their venoms which may interfere with host development and immune response [9].

## Conclusions

The galling habit represents a complex type of interaction between insects and plants, ranging from antagonism to mutualism, as is the case for *Ficus* plants. However, the molecular and chemical signals involved in gall induction in this and other systems are poorly known.

This study sheds light on the understanding of species interactions from a molecular perspective. We showed that venoms from fig wasps are mainly constituted of peptides and proteins, which brings a new perspective to the investigation of gall-inducing molecules, so far deeply focused on phytohormones. We also show that venom protein fingerprints from galling fig wasps differ from the venom fingerprints of non-galling species, suggesting the secretion plays different roles according to the type of interaction it is involved in. The differences in venom composition observed between galling and non-galling fig wasp species bring new perspectives to the study of gall induction processes and the role of insect secretions.

## Supporting information

S1 Fig Studied fig wasp species. a- *Ceratosolen solmsi* (pollinating ovary-galling species); b- *Pegoscapus* sp. (pollinating ovary-galling species); c- *Sycophaga* sp. (non—pollinating ovary-galling species); d- *Idarnes* sp. 3 (non—pollinating ovary-galling species); e- *Philotrypesis pilosa* (cleptoparasite); f- *Idarnes* sp. 1 (cleptoparasite); g- *Sycoryctes* aff. *trifemmensis* (parasitod). Scale bar = 500μm.
(PDF)

S2 Fig Mass spectra obtained by MALDI-TOF MS (linear positive ion mode) from three different samples (each sample consists of a pool of 10 reservoirs) of the galling wasp *Pegoscapus aerumnosus*. (PDF)

S3 Fig Mass spectra obtained by MALDI-TOF MS (linear positive ion mode) from reservoirs of the galling wasp *Pegoscapus* sp. Analyses were carried out immediately after dissection (A) and after 6 weeks of storage at 2–6°C (B).
(PDF)

S4 Fig Mass spectrum obtained by MALDI-TOF (reflector positive ion mode) from reservoirs of the non-galling wasp *Sycoryctes* aff. trifemmensis.
(PDF)

S5 Fig Mass spectrum obtained by MALDI-TOF (linear positive ion mode) from reservoirs of the non-galling wasp *Sycoryctes* aff. *trifemmensis*.
(PDF)

S6 Fig Partial Least Squares Discriminant Analysis based on the intensity matrix of ions detected by MALDI-TOF MS in the 1–20 KDa range. Samples from pollinating ovary-galling wasps are represented by cubes (*Pegoscapus* sp. in light blue and *Ceratosolen solmsi* in pink). Samples from non-pollinating ovary-galling wasps are represented by tetrahedrons (*Idarnes* sp. 3 in black and *Sycophaga* sp.in green). Non-galling cleptoparasites samples are represented by icosahedrons (*Idarnes* sp. 1 in grey and *Philotrypesis pilosa* in brown). The non-galling parasitod *Sycoryctes* aff. *trifemmensis* samples are represented by blue spheres.
(PDF)

S7 Fig Ranking of the ten most important discriminant peaks in the partial least squares discriminant analysis. Analysis was based on the intensity matrix of ions detected from venom reservoir samples by MALDI-TOF MS in the 1–20 KDa range.
(PDF)

S8 Fig Total ion chromatogram of venom reservoirs obtained using GC-MS. Spectra correspond to non-galling wasp *Idarnes* sp. 1 (A), galling wasp *Idarnes* sp. 3 (B) and the solvent, which was used as control (C).
(PDF)

S9 Fig Total ion chromatogram of venom reservoirs obtained using UPLC-ESI MS in negative ion mode. Spectra correspond to galling wasp *Idarnes* sp. 3 (A), non-galling wasp *Idarnes* sp. 1 (B) and the solvent, which was used as control (C).
(PDF)

S10 Fig Extracted ion chromatogram of ion *m/z* 174 [M-H]^-^ (relative to indole-3-acetic acid) obtained using UPLC-ESI MS in negative ion mode. Spectra correspond to venom reservoirs from galling wasp *Idarnes* sp. 3 (A), venom reservoirs from non-galling wasp *Idarnes* sp. 1 (B) and the solvent, which was used as control (C).
(PDF)

S11 Fig Extracted ion chromatogram of ion *m/z* 220 [M+H]^+^ obtained using UPLC-ESI MS in positive ion mode. Spectra correspond to venom reservoirs from galling wasp *Idarnes* sp. 3 (A), venom reservoirs from non-galling wasp *Idarnes* sp. 1 (B) and the solvent, which was used as control (C).
(PDF)

S12 Fig Extracted ion chromatogram of ion *m/z* 204 obtained using UPLC-ESI MS in positive ion mode. Spectra correspond to venom reservoirs from galling wasp *Idarnes* sp. 3 (A), venom reservoirs from non-galling wasp *Idarnes* sp. 1 (B) and the solvent, which was used as control (C).
(PDF)

S13 Fig Extracted ion chromatogram of ion *m/z* 336 obtained using UPLC-ESI MS in positive ion mode. Spectra correspond to venom reservoirs from galling wasp *Idarnes* sp. 3 (A), venom reservoirs from non-galling wasp *Idarnes* sp. 1 (B) and the solvent, which was used as control (C).
(PDF)
