# Supplementary material for: A comparative venomic fingerprinting approach reveals that galling and non-galling fig wasp species have different venom profiles
Source: PLoS One. 2018 Nov 8;13(11):e0207051. doi: 10.1371/journal.pone.0207051 (PMC6224076; doi:10.1371/journal.pone.0207051)
Supplement: S1 Fig — a- Ceratosolen solmsi (pollinating ovary-galling species); b- Pegoscapus sp. (pollinating ovary-galling species); c- Sycophaga sp. (non—pollinating ovary-galling species); d- Idarnes sp. 3 (non—pollinating ovary-galling species); e- Philotrypesis pilosa (cleptoparasite); f- Idarnes sp. 1 (cleptoparasite); g- Sycoryctes aff. trifemmensis (parasitod). Scale bar = 500μm. (PDF) [file pone.0207051.s001.pdf]

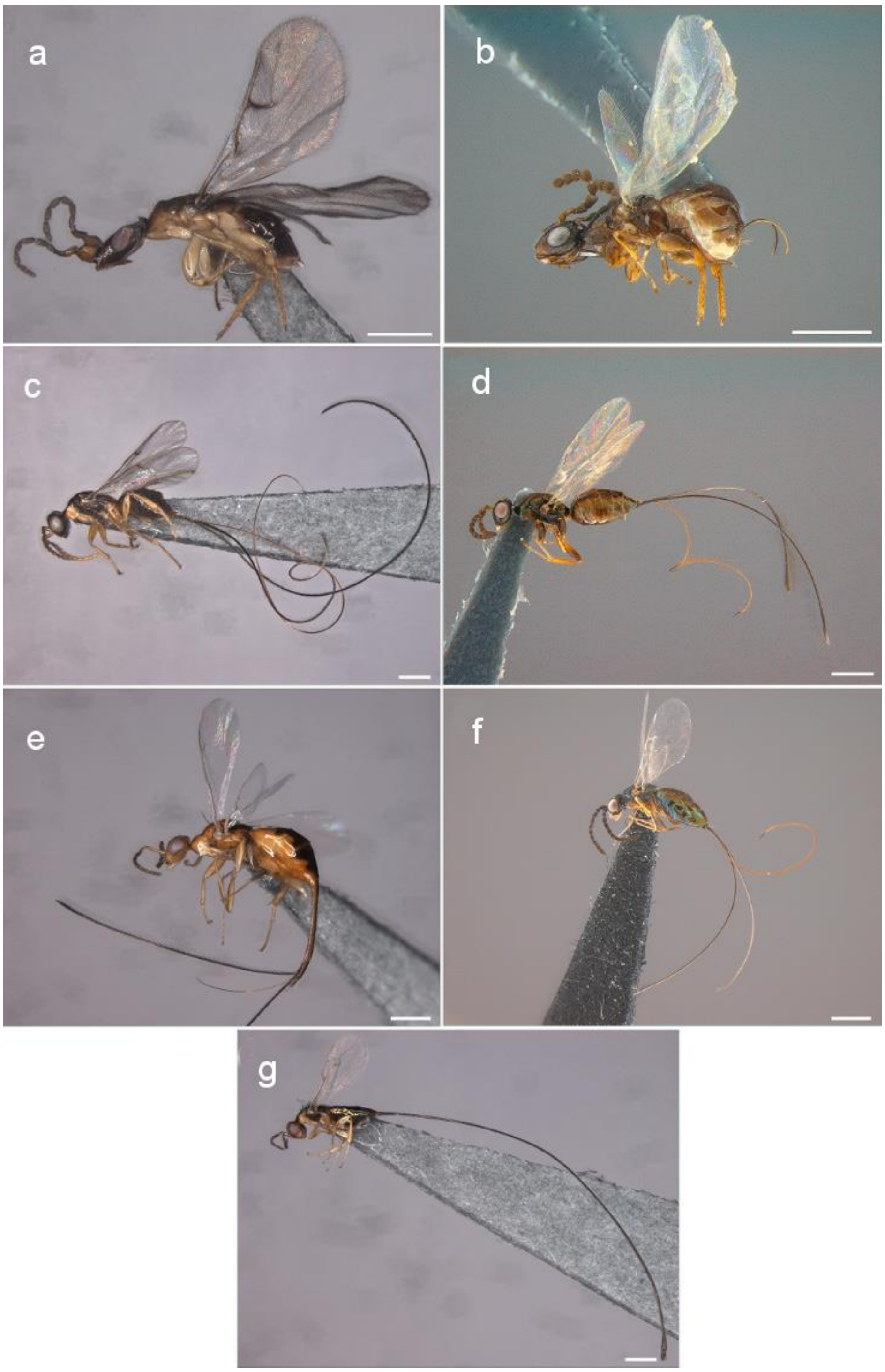

Supplemental Figure S1| Studied fig wasp species. a- *Ceratosolen solmsi* (pollinating ovary-galling species); b- *Pegoscapus aerumnosus* (pollinating ovary-galling species); c- *Sycophaga* sp. (non - pollinating ovary-galling species); d- *Idarnes* sp. 3 (non - pollinating ovary-galling species); e- *Philotrypesis pilosa* (cleptoparasite); f- *Idarnes* sp. 1 (cleptoparasite); g- *Sycoryctes* aff. *trifemmensis* (parasitod). Scale bar = 500µm.
