# Supplementary material for: A comparative venomic fingerprinting approach reveals that galling and non-galling fig wasp species have different venom profiles
Source: PLoS One. 2018 Nov 8;13(11):e0207051. doi: 10.1371/journal.pone.0207051 (PMC6224076; doi:10.1371/journal.pone.0207051)
Supplement: S2 Fig — (PDF) [file pone.0207051.s002.pdf]

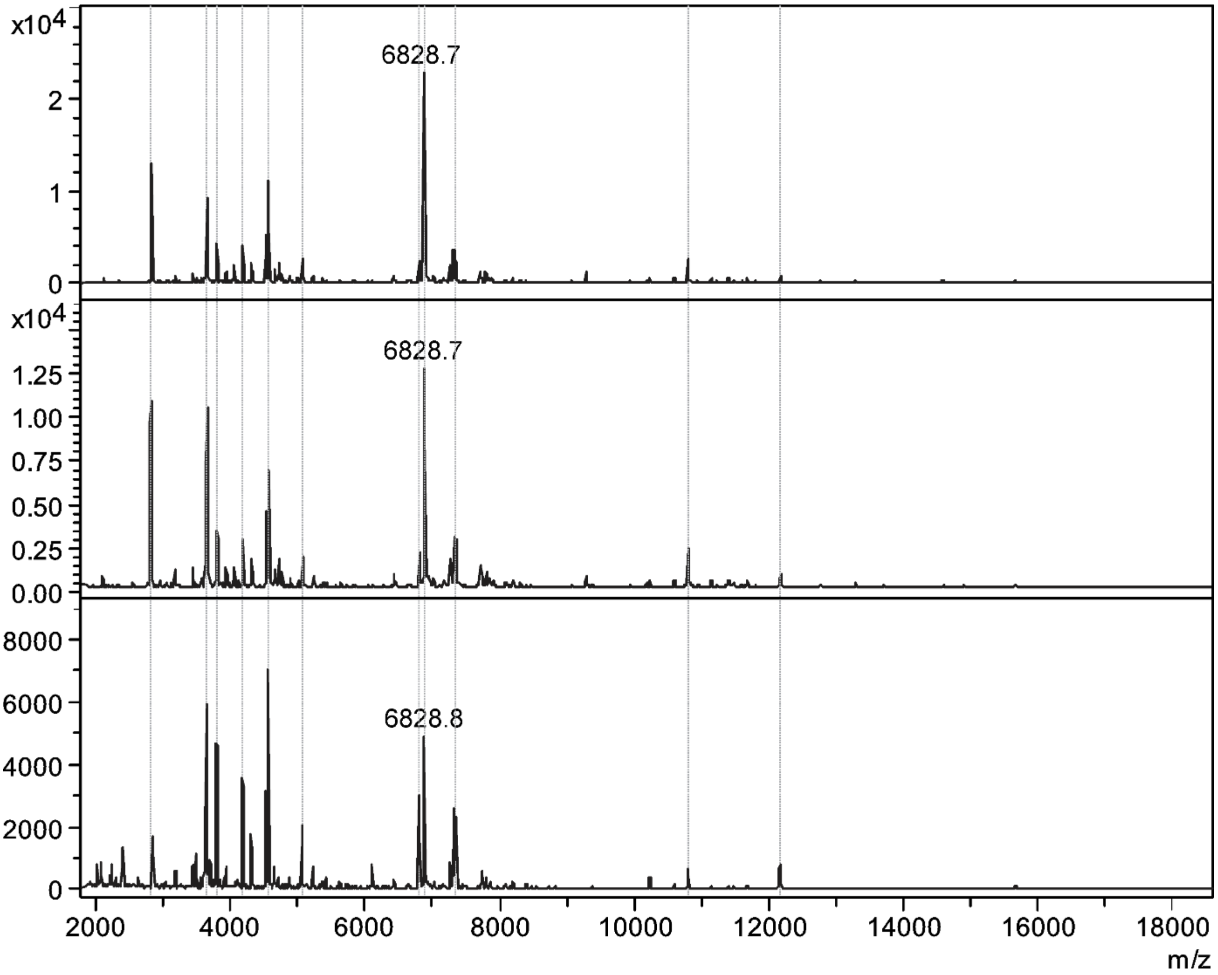

Supplemental Figure S2| Mass spectra obtained by MALDI-TOF MS (linear positive ion mode) from three different samples (each sample consists of a pool of 10 reservoirs) of the galling wasp *Pegoscapus aerumnosus*.
