# Supplementary material for: A comparative venomic fingerprinting approach reveals that galling and non-galling fig wasp species have different venom profiles
Source: PLoS One. 2018 Nov 8;13(11):e0207051. doi: 10.1371/journal.pone.0207051 (PMC6224076; doi:10.1371/journal.pone.0207051)
Supplement: S3 Fig — Analyses were carried out immediately after dissection (A) and after 6 weeks of storage at 2–6°C (B). (PDF) [file pone.0207051.s003.pdf]

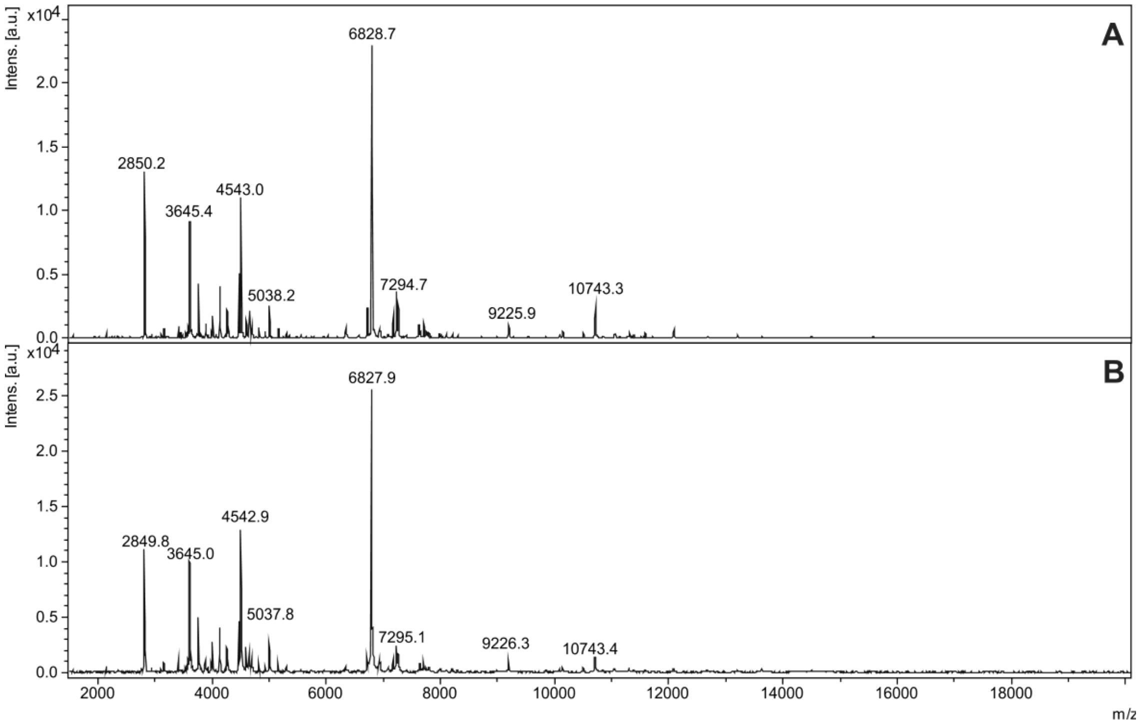

Supplemental Figure S3| Mass spectra obtained by MALDI-TOF MS (linear positive ion mode) from reservoirs of the galling wasp *Pegoscapus aerumnosus* analysed immediately after dissection (A) and after 6 weeks of storage at 2-6 ºC (B).
