# Supplementary material for: A comparative venomic fingerprinting approach reveals that galling and non-galling fig wasp species have different venom profiles
Source: PLoS One. 2018 Nov 8;13(11):e0207051. doi: 10.1371/journal.pone.0207051 (PMC6224076; doi:10.1371/journal.pone.0207051)
Supplement: S5 Fig — trifemmensis. (PDF) [file pone.0207051.s005.pdf]

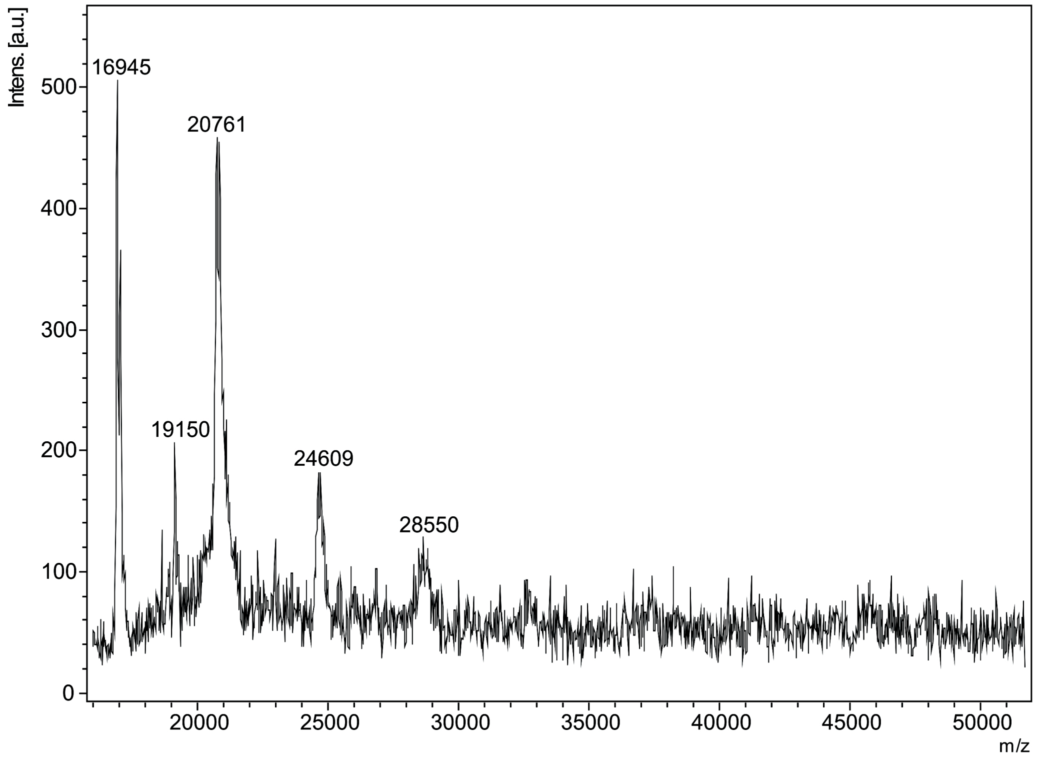

Supplemental Figure S5| Mass spectrum obtained by MALDI-TOF (linear positive ion mode) from reservoirs of the non-galling wasp *Sycoryctes* aff. *trifemmensis*.
