# Supplementary material for: A comparative venomic fingerprinting approach reveals that galling and non-galling fig wasp species have different venom profiles
Source: PLoS One. 2018 Nov 8;13(11):e0207051. doi: 10.1371/journal.pone.0207051 (PMC6224076; doi:10.1371/journal.pone.0207051)
Supplement: S6 Fig — Samples from pollinating ovary-galling wasps are represented by cubes (Pegoscapus sp. in light blue and Ceratosolen solmsi in pink). Samples from non-pollinating ovary-galling wasps are represented by tetrahedrons (Idarnes sp. 3 in black and Sycophaga sp.in green). Non-galling cleptoparasites samples are represented by icosahedrons (Idarnes sp. 1 in grey and Philotrypesis pilosa in brown). The non-galling parasitod Sycoryctes aff. trifemmensis samples are represented by blue spheres. (PDF) [file pone.0207051.s006.pdf]

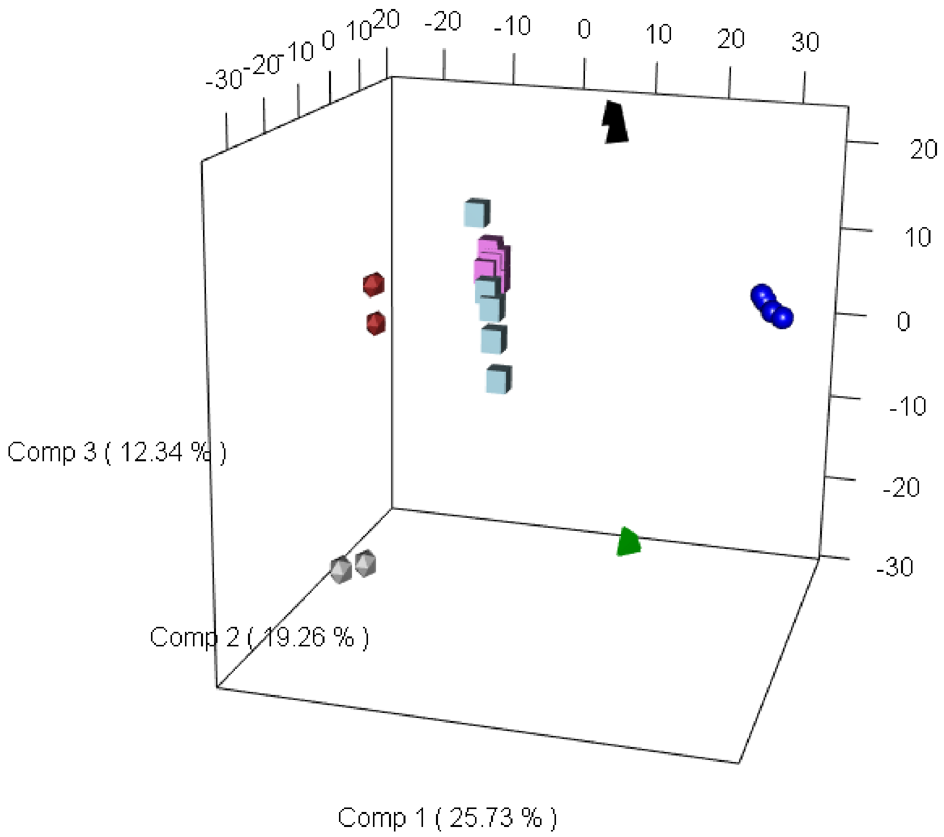


**Supplemental Figure S6|** Partial Least Squares Discriminant Analysis based on the intensity matrix of ions detected by MALDI-TOF MS in the 1-20 KDa range. Samples from pollinating ovary-galling wasps are represented by cubes (*Pegoscapus aerumnosus* in light blue and *Ceratosolen solmsi* in pink). Samples from non-pollinating ovary-galling wasps are represented by tetrahedrons (*Idarnes* sp. 3 in black and *Sycophaga* sp.in green). Non-galling cleptoparasites samples are represented by icosahedrons (*Idarnes* sp. 1 in grey and *Philotrypesis pilosa* in brown). The non-galling parasitod *Sycoryctes* aff. *trifemmensis* samples are represented by blue spheres.
