# Supplementary material for: A comparative venomic fingerprinting approach reveals that galling and non-galling fig wasp species have different venom profiles
Source: PLoS One. 2018 Nov 8;13(11):e0207051. doi: 10.1371/journal.pone.0207051 (PMC6224076; doi:10.1371/journal.pone.0207051)
Supplement: S7 Fig — Analysis was based on the intensity matrix of ions detected from venom reservoir samples by MALDI-TOF MS in the 1–20 KDa range. (PDF) [file pone.0207051.s007.pdf]

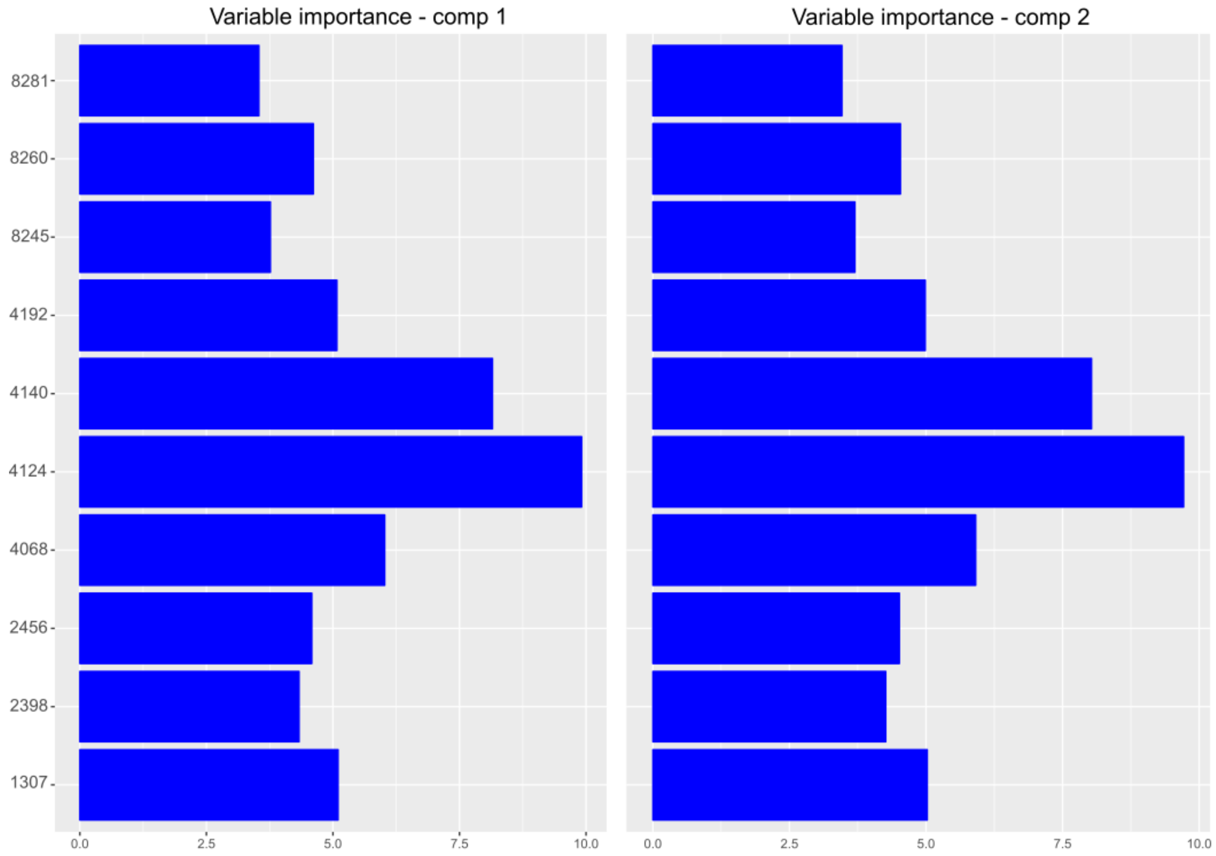

Supplemental Figure S7| Ranking of the ten most important discriminant peaks in the partial least squares discriminant analysis. Analysis was based on the intensity matrix of ions detected from venom reservoir samples by MALDI-TOF MS in the 1-20 KDa range.
