# Supplementary material for: A comparative venomic fingerprinting approach reveals that galling and non-galling fig wasp species have different venom profiles
Source: PLoS One. 2018 Nov 8;13(11):e0207051. doi: 10.1371/journal.pone.0207051 (PMC6224076; doi:10.1371/journal.pone.0207051)
Supplement: S13 Fig — Spectra correspond to venom reservoirs from galling wasp Idarnes sp. 3 (A), venom reservoirs from non-galling wasp Idarnes sp. 1 (B) and the solvent, which was used as control (C). (PDF) [file pone.0207051.s013.pdf]

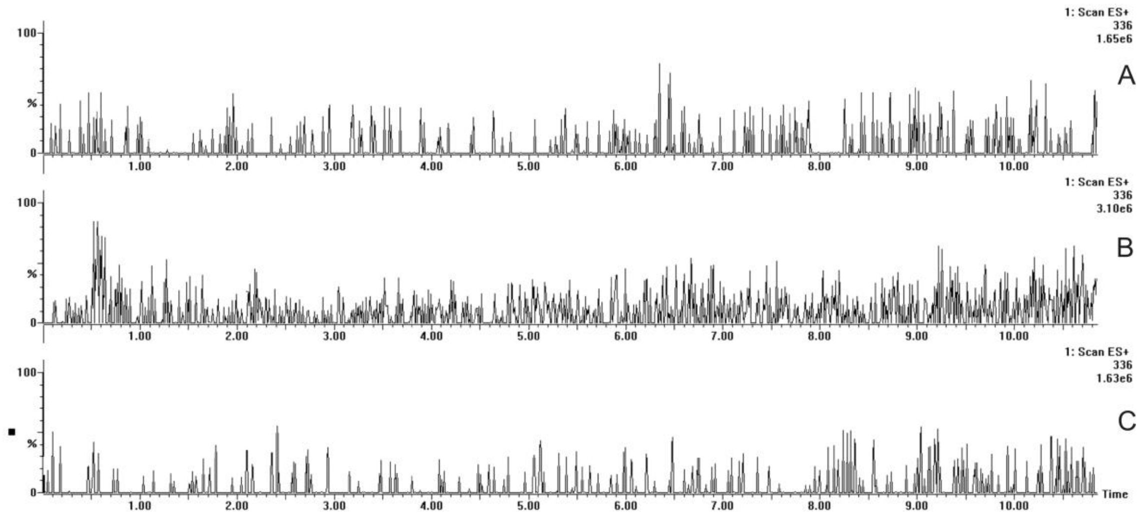

Supplemental Figure S13| Extracted ion chromatogram of ion *m/z* 336 obtained using UPLC-ESI MS in positive ion mode. Spectra correspond to venom reservoirs from galling wasp *Idarnes* sp. 3 (A), venom reservoirs from non-galling wasp *Idarnes* sp. 1 (B) and the solvent, which was used as control (C).
